# Exomic Sequencing of Immune-Related Genes Reveals Novel Candidate Variants Associated with Alopecia Universalis

**DOI:** 10.1371/journal.pone.0053613

**Published:** 2013-01-11

**Authors:** Seungbok Lee, Seung Hwan Paik, Hyun-Jin Kim, Hyeong Ho Ryu, Soeun Cha, Seong Jin Jo, Hee Chul Eun, Jeong-Sun Seo, Jong-Il Kim, Oh Sang Kwon

**Affiliations:** 1 Genomic Medicine Institute (GMI), Medical Research Center, Seoul National University, Seoul, Korea; 2 Department of Biomedical Sciences, Seoul National University Graduate School, Seoul, Korea; 3 Department of Dermatology, Seoul National University College of Medicine, Seoul, Korea; 4 Laboratory of Cutaneous Aging and Hair Research, Clinical Research Institute, Seoul National University Hospital, Seoul, Korea; 5 Institute of Dermatological Science, Seoul National University College of Medicine, Seoul, Korea; 6 Department of Biochemistry and Molecular Biology, Seoul National University College of Medicine, Seoul, Korea; 7 Psoma Therapeutics Inc., Seoul, Korea; 8 Macrogen Inc., Seoul, Korea; Ohio State University Medical Center, United States of America

## Abstract

Alopecia areata (AA) is a common autoimmune disorder mostly presented as round patches of hair loss and subclassified into alopecia totalis/alopecia universalis (AT/AU) based on the area of alopecia. Although AA is relatively common, only 5% of AA patients progress to AT/AU, which affect the whole scalp and whole body respectively. To determine genetic determinants of this orphan disease, we undertook whole-exome sequencing of 6 samples from AU patients, and 26 variants in immune-related genes were selected as candidates. When an additional 14 AU samples were genotyped for these candidates, 6 of them remained at the level of significance in comparison with 155 Asian controls (p<1.92×10^−3^). Linkage disequilibrium was observed between some of the most significant SNPs, including rs41559420 of *HLA-DRB5* (p<0.001, OR 44.57) and rs28362679 of *BTNL2* (p<0.001, OR 30.21). While *BTNL2* was reported as a general susceptibility gene of AA previously, *HLA-DRB5* has not been implicated in AA. In addition, we found several genetic variants in novel genes (*HLA-DMB*, *TLR1*, and *PMS2*) and discovered an additional locus on *HLA-A*, a known susceptibility gene of AA. This study provides further evidence for the association of previously reported genes with AA and novel findings such as *HLA-DRB5*, which might represent a hidden culprit gene for AU.

## Introduction

Alopecia areata (AA) is characterized by autoimmune-mediated patchy hair loss. The alopetic patches can be multiple and coalesce to diffuse hair loss over the scalp, even the whole body. Severe forms of AA are named based on the body area affected by alopecia and include alopecia totalis (AT; affecting the whole scalp) and alopecia universalis (AU; affecting the entire body). Although AA is fairly common, with the life-time risk being 1.7% [1], progression to AT/AU is rare and only reported in 5% of AA patients [2]. Clinically, the extent of hair loss is the most important prognostic factor in AA [3]. Although more than 50% of classic AA patients recover without treatment [4], less than 10% of patients with AT/AU recover fully [5].

The underlying etiopathogenesis of AA has been revealed as an autoimmune mechanism from multiple lines of studies. Histologically, AA is characterized by immune infiltrates surrounding the hair follicle. Moreover, AA is responsive to immunosuppressive therapies, further supporting an autoimmune basis for the disease [6]. In addition, several genetic studies on AA have shown that immune factors are key genetic components of AA. Early association studies with selected candidate genes revealed that AA or AT/AU is associated with several immune-related genes including *HLA-DQB1*, *MICA*, *PTPN22*, *IL1RN*, *AIRE*, and *TRAF1/C5* [7]–[14]. Furthermore, *HLA-DQB1*, *MICA*, *PTPN22*, *IL1RN*, and *TRAF1/C5* were found to influence disease severity [7], [9]–[11], [13], [14]. Genetic susceptibility loci harboring murine homologues of *HLA* have also been discovered in a C3H/HeJ mouse model of AA [15]. In addition, recent genome-wide linkage and association studies have identified several susceptibility loci near, or within, genes related to autoimmunity, such as *MICA* and *BTNL2* [16]–[18].

Although previous studies have identified AA as an immune-related disorder and have shown several genetic loci throughout the genome, association studies for the most severe type of AA (AU) have rarely been performed, especially using next-generation sequencing technology. Most rare missense genetic variations are predicted to be deleterious and, thus, association studies with these variants may be helpful in this regard [19]. Recent studies have also suggested that the majority of variants seem to be rare, and these rare variants are likely to be key determinants for the health status of individuals [20], [21]. Given that AU patients are rare and have the most severe AA phenotype, identification of rare variants with large effect sizes would be a good strategy to further elucidate the genetic basis for this disease.

In this study, on the basis of the immune-related mechanism of the disease, we conducted an exomic sequence analysis of immune-related genes in AU patients with the aim of identifying genetic determinants of AU by focusing on rare and pathogenic candidates.

## Materials and Methods

### Ethics statement

Ethical approval for the study was obtained from the relevant Ethics Committees (The Institutional Review Board of the Seoul National University Hospital). This study was conducted according to Declaration of Helsinki principles. Written informed consent was obtained before blood was taken from patients with AU.

### Study sample selection and DNA extraction

For this study, 20 patients with AU were recruited from the outpatient clinic of Seoul National University Hospital. Overall demographic data and clinical features are summarized in Table 1, and detailed individual data are presented in Table S1. A diagnosis of AU was based on any experience of whole body hair loss in the participant's lifetime, and involved areas other than the scalp were eyebrow, axillary hair, and pubic hair (eyebrow only in child). Genomic DNA was extracted from peripheral blood by using QuickGene DNA whole blood kit with QuickGene-610L equipment (Fujifilm, Tokyo, Japan). DNA concentrations were measured by Nanodrop ND-1000 spectrophotometer (Nano-drop Technologies, Delaware, USA).

**Table 1:** Demographic data of enrolled patients.

**Age, years**		3–43 (mean: 15)
**Gender, n**	Male	13 (65%)
	Female	7 (35%)
**Onset, n** 1	Early onset	15 (75%)
	Late onset	5 (25%)
**Disease duration, years**		1–18 (mean: 5.5)
**Nail dystrophy, n**		6 (30%)
**Family history, n**		6 (30%)
**Comorbid disorders, n**	Atopy	2 (10%)
	Thyroid disease	2 (10%)
	Psoriasis	1 (5%)

1 Based on the age at onset of < or ≥13 yr, patients were classified to the early-onset group.

### Whole-exome sequencing of 6 individuals with alopecia universalis

Exomic regions of DNA were captured by SureSelect Human All Exon v.2 Kits (Agilent Technologies, California, USA) and sequenced through HiSeq 2000 (Illumina, California, USA) in 6 samples among study subjects. All procedures followed the manufacturer's instructions. The generated reads were aligned to the NCBI human reference genome build 37 (hg19) using the Genomic Short-read Nucleotide Alignment Program alignment tool [22]. Only positions having ≥8 uniquely aligned reads and ≥20 mean quality score (Q score) were carried forward for further analyses. Variant calling was performed according to the criteria of our previous study [23], and all the variants called were annotated by the RefSeq gene set and compared with dbSNP build 132.

### Filtration into functional candidate variants

In this study, we focused on the variants found in immune-related genes. Candidate genes were categorized by gene ontology by using DAVID [24] and only variants in immune-related genes were collected. Among them, we selected nonsynonymous single nucleotide polymorphisms (SNPs), coding sequence insertions/deletions (indels), and canonical splice-site variants as pathogenic candidates.

For further filtration, genotype frequencies of pathogenic candidates were compared between patient and control groups in both dominant and recessive models. The control samples used for this study were as follows: (1) 59 Korean samples (8 from whole-genome sequencing (WGS), 41 from whole-exome sequencing (WES)), (2) 56 Asian HapMap samples (10 from WGS, 46 from WES), and (3) 40 Mongolian samples from WES. These controls were selected from the Asian Genome Project of our group, and 58 of them were published in our previous studies [25], [26]. Statistical comparisons were drawn using a non-parametric method of Fisher's exact test, and a p-value less than 0.05 was used as a standard for the candidate selection. To reduce the number of variants selected by chance, we further narrowed our candidates into those shown in at least 2 patient samples.

Finally, we manually checked the coverage depth of each variant and excluded variants showing deviated patterns of coverage. The deviated pattern of coverage was defined as a coverage depth ratio between the reference and variant alleles that is either >0.5 or <0.5 throughout the samples (Table S2).

Overall filtration steps above were summarized in Figure S1. In addition to the candidates selected, rs28362679 in *BTNL2* was included since it is not only associated with MHC class II but also previously highlighted as a susceptibility gene for the development of AA [17].

### Further genotyping in 14 additional alopecia universalis patients and frequency comparison with control samples

The remaining variants were genotyped in the other 14 patient samples with PCR and subsequent Sanger sequencing (Table S3). Using Fisher's exact test, the genotype frequency of each variant was compared with that of 155 control samples in both dominant and recessive models. For both analyses, the threshold level of significance was set at a p-value lower than 1.92×10^−3^, considering multiple testing of our selected candidates (n = 26). For reference, the allele frequency of each variant in Asians was also checked against the 1000 Genomes Project by using the ENGINES program [27].

### Linkage disequilibrium analysis

We explored the linkage disequilibrium (LD) structures among final candidates to check if there were any crucial haplotypes associated with AU. The genotypes of all 20 patient samples were used for LD estimation, which was calculated by the Haploview program (Version 4.2). We defined LD structure between SNPs when an r^2^ value was higher than 0.3, and pairwise comparisons of markers >500 kb apart were ignored.

## Results

We sequenced exomic regions of 6 individuals with AU and aligned the generated reads to the NCBI human reference genome build 37 (Table 2). The percentage of covered bases in the bait region was 84.7% on average (76.2–88.75%) with the mean coverage depth of 69.1× (43.6–100.0×). SNPs and short indels were called according to the criteria set out in our previous study, and annotated based on the RefSeq gene set and dbSNP build 132 [23]. The annotated SNPs and indels were filtered stepwise as described in the methods, and narrowed down to 25 SNPs and 1 indel by excluding variants showing ambiguous patterns of coverage depth in exome sequencing and a variant with PCR failure (Table 3).

**Table 2:** Sequencing summary of patient samples.

Samples	Total reads	Uniquely aligned reads	% Covered bait bases^1^ (≥1)	% Covered bait bases (≥8)	Mean coverage depth in bait regions (x)
AU01	65,959,668	57,329,095	96.07	87.25	58.65
AU02	41,309,992	35,686,769	94.06	79.73	35.32
AU03	71,009,718	61,311,833	96.23	87.72	62.09
AU04	53,085,782	46,056,902	95.58	84.9	46.53
AU05	74,426,242	64,677,337	96.39	88.34	65.39
AU06	121,993,380	75,311,600	89.23	76.22	76.66

1 Bases within the bait region defined as the region covered by more than one capture probe.

**Table 3:** Association between alopecia universalis and selected markers in the case-control analysis.

			Dominant		Recessive			
Variant	Gene	Risk allele	p-value	OR	p-value	OR	1000 Genomes^1^	NGS Controls^2^
rs41559420	*HLA-DRB5*	A	<0.001	44.571	<0.001	-	0.014	0.005
rs28362679	*BTNL2*	A	<0.001	30.214	-	-	0.021	0.007
rs113337937	*HLA-A*	del	<0.001	18.857	-	-	-	0.088
rs116375983	*HLA-DMB*	T	<0.001	8.917	0.159	3.861	-	0.14
rs1805323	*PMS2*	T	0.001	8.125	0.332	1.683	0.348	0.31
rs117033348	*TLR1*	G	0.001	7.754	-	-	0.014	0.032
rs1126477	*LTF*	T	0.002	6.847	0.193	2.19	0.4	0.292
rs2277244	*DMBT1*	T	0.003	6.074	-	-	0.079	0.049
rs2297950	*CHIT1*	T	0.006	4.024	0.017	7.667	0.295	0.174
rs17130745	*GBP4*	C	0.009	6.083	0.115	-	0.033	0.026
rs78108426	*CIITA*	A	0.009	4.667	-	0	0.131	0.069
rs41542812	*HLA-DQB1*	G	0.015	5.4	0.01	-	0.058	0.037
rs75268750	*C5*	A	0.019	4	-	-	0.072	0.048
rs161704	*IL31RA*	A	0.023	3.16	0.18	2.156	0.358	0.295
rs2276872	*CD96*	C	0.024	3.025	0.387	2.667	0.157	0.116
rs17847215	*INPPL1*	C	0.036	4	-	-	0.075	0.048
rs2273346	*MASP2*	G	0.046	2.522	0.081	4.059	0.175	0.184
rs2738047	*DEFB1*	T	0.053	5.153	-	-	0.01	0.017
rs4304840	*CLEC4D*	G	0.06	2.413	-	0	0.154	0.14
rs116306892	*MICA*	C	0.084	2.227	0.069	4.553	-	0.153
rs34531670	*KIR3DP1*	C	0.106	2.114	0.248	2.593	-	0.14
rs4911290	*C20orf185*	A	0.223	3.2	0.494	1.143	0.248	0.288
rs116897146	*KIR3DP1*	C	0.226	1.66	0.541	1.495	-	0.139
rs1799945	*HFE*	G	0.289	2.254	-	-	0.024	0.023
rs1049107	*HLA-DQB1*	T	0.3	1.548	0.094	3.625	-	0.161
rs17611	*C5*	T	0.393	1.404	0.024	2.949	0.418	0.497

Abbreviations: OR, odds ratio; SNP, single nucleotide polymorphism; MAF, minor allele frequency; del, deletion; NGS, next-generation sequencing.

1 Minor allele frequency of each variant in 1000 Genomes Phase I [27].

2 Minor allele frequency of each variant in 155 controls used in this study.

Next, we genotyped the final 26 candidates in additional 14 AU samples by using PCR and subsequent Sanger sequencing method. The frequency of each candidate was compared with that of control samples in both dominant and recessive models (Table 3). As a result, 6 out of 26 candidates had p-values less than 1.92×10^−3^. Although all these variants were novel as AA or AT/AU susceptibility variants, *BTNL* and *HLA-A* had previously been reported as genetic candidates for the development of AA [17], [28].

To determine whether there were any haplotypes composed within our candidate variants, we explored the LD structure among them by using genotype data from our AU patients (Table 4). As a result, we found 2 LD structures among 26 candidates; one included rs28362679, rs41559420, rs41542812, and rs1049107, whilst the other included rs116897146 and rs34531670 (r^2^>0.3). The first haplotype (unlike the second) contained some significant SNPs, including rs41559420 of *HLA-DRB5* (p<0.001, OR 44.57) and rs28362679 of *BTNL2* (p<0.001, OR 30.21).

**Table 4:** Linkage disequilibrium estimation among candidates (r^2^>0.3).

Chromosome	Variant 1	Variant 2	r^2^	D′
6	rs28362679	rs41559420	0.41	1
		rs41542812	0.61	1
		rs1049107	0.47	1
	rs41559420	rs41542812	0.68	1
		rs1049107	0.49	0.74
	rs41542812	rs1049107	0.77	1
19	rs116897146	rs34531670	0.75	1

The *HLA-DQB1* and *C5* genes have been suggested as susceptibility genes of AA previously [7], [13]. Even though rs41542812 of *HLA-DQB1* (p = 0.015, OR 5.4) and *C5* (p = 0.019, OR 4.0) did not reach the significance level of this study, studies with larger sample sizes may alter the decisions for these variants. In addition, involvement of the *TRAF1/C5* locus in the etiology of familial and severe AA was revealed in a recent study [13].

## Discussion

Our study revealed several immune-related susceptibility variants for AU and the LD between some of the most significant variants. Of the 2 statistically significant genes in the associated combination of alleles, *BTNL2* has already been associated with the development of AA [17], while *HLA-DRB5* has not previously been implicated in the pathogenesis of this disease. The *HLA-DRB5* gene encodes the major histocompatibility complex, class II, DR beta 5. This class II molecule plays an important role in the immune system by presenting antigens in antigen-presenting cells. The presence of a certain HLA gene might ensure more proficient presentation of a particular autoantigen and, consequently, the association of HLA genes with autoimmune diseases has received a great deal of attention. The associations of *HLA-DRB5* with autoimmune disorders such as multiple sclerosis [29], systemic sclerosis [30], and rheumatic heart disease [31] have been reported. Given the assumed autoimmune development of AA, we hypothesized that *HLA-DRB5* might also be responsible for the development of AA.

Although numerous previous studies including 2 genome-wide association studies (GWAS) have been performed, the association of *HLA-DRB5* with AA has not yet been reported to our knowledge. In this study, however, the nonsynonymous SNP (rs41559420) in *HLA-DRB5* was most significant in the comparative analysis of 20 AU patients with controls, showing the highest odds ratio among candidates (Table 3). This may indicate the existence of different pathological mechanisms between AU and AA, or these kinds of variants may have been missed, or not reported, in the previous studies because of their considerable rarity. Indeed, the Illumina 610 K and 550 K, used in the previous 2 GWAS studies performed on AA, do not contain this SNP [17], [18]. Even if these arrays did include it, it would not have been analyzed since SNPs with low minor allele frequency (MAF) are usually excluded during the quality control process (Table 3, see the allele frequencies in the columns of MAF).

In addition to *BTNL2*, we also replicated another gene, *HLA-A*, to be associated with AA at the gene level [28]. Besides these, other variants found in this study were all located in novel genes, including *HLA-DMB*, *PMS2*, and *TLR1*. The *HLA-DMB* gene encodes HLA class II beta chain paralogue like *HLA-DRB5* [32] and this molecule is an important component of the adaptive immune system. *TLR1*, which plays fundamental roles in innate immunity [33], [34], was also first reported in AA. Since both adaptive and innate immunoregulatory genes are known to be involved in the pathogenesis of AA [17], these novel findings warrant further research. Mutations in *PMS2* are important in the progression of human cancer [35], [36], and deficiency of human PMS2 is associated with impaired immunoglobulin class switch recombination [37]. The association of cancer immunology components with AA requires further validation.

In Table S4, we list all the pathogenic candidates satisfying the candidate selection criteria of this study, regardless of gene ontology, for the researchers interested in AA or AT/AU. Even if only the immune-related variants were selected for this study, there could be more explanations on disease development other than autoimmunity, or unknown genes participating in the immune system. Furthermore, as AA or AT/AU is a well-known complex disorder determined by multiple genetic and environmental factors, these listed variant would be open to a diversity of interpretations when more patient samples are sequenced or various studies are conducted on different points of view.

Genes involved in the hair development can be good examples of such possible candidates for the AU pathogenesis, other than immune-related genes. Previous studies have reported several genes for hair development, such as *HR*, *LHX2* [38], and genes involved in Wnt signaling [39], Sonic hedgehog (Shh) signaling [40], and bone morphogenetic protein (BMP) signaling pathways [41]. Among the pathogenic candidates in Table S4, we found 3 variants in Wnt signaling genes, including *AXIN2*, *POSTN*, and *VCAN*. Although these were not included for this study, additional research might determine their roles in AU pathogenesis.

We acknowledge that the sample size in our study is relatively small, and only 6 samples were sequenced by next-generation sequencing technology. Small sample sizes have higher probability for false positives, which accidentally co-occur in case samples. However, given that causative variants in these extreme phenotypes are likely to be quite rare but may have large effect sizes, targeted and stepwise approaches may minimize this limitation. First, as the pathogenesis of AA is thought to be autoimmune, only variants in immune-related genes were concerned for further validation study. Second, we selected candidate variants that could affect the functions of the encoded proteins through altering amino acid sequences. Third, we selected candidate variants shown in at least 2 samples and manually checked the coverage depth of each position in an attempt to increase reliability and reduce the false positive calls. Throughout these filtration steps, our study could select several candidate variants from exome sequencing results and successfully validate 6 of them in our study subjects.

This study has another limitation for suggesting decisive candidates for AU development; a lack of replication. However, 14 samples, which were additionally sequenced for final analyses, were independent from the first 6 samples. When these 14 samples were analyzed separately, 8 candidates, including the 6 variants which were significant at last, showed similar levels of significance with those in the candidate selection analysis (p<0.05, Table S5). These results enhance the possibility that our candidates might be causative for AU pathogenesis, even though future studies on similar or different populations are necessary to confirm our findings.

To identify genetic determinants in patients with the most severe AA phenotype, we conducted an association study after completion of WES and discovered candidate variants by using a stepwise filtering approach. Through the WES approach, several studies have successfully discovered the causative genes in rare Mendelian disorders [42], [43]. Furthermore, exome sequencing followed by a subsequent association study was recently performed in a small population with extreme phenotypes and showed the utility of next-generation sequencing in searching for causative variants in complex traits [44]. Now, due to the advance of high-throughput sequencing technologies, it is predicted that these approaches will potentially replace array-based technologies and open up a new era of genetic research [45]. The current findings are another example of the potential use of sequencing technologies to identify causative candidate genes in complex disorders.

In conclusion, through an exomic sequence analysis on immune-related genes, we found several candidate variants and haplotypes associated with AU. This study provides further evidence for the involvement of immune-related genes in the development of AA and identifies novel candidates such as *HLA-DRB5*, which may play a previously unrecognized role in the pathogenesis of the disease.

## Supporting Information

Figure S1 **The flow of filtration steps used for candidate variant selection.**
(PPTX)

Table S1 **Demographic data and clinical presentations of study subjects.**
(DOC)

Table S2 **Base coverage and Q-score of each variant in 155 controls and 6 cases.**
(XLS)

Table S3 **Primer pairs for genotyping of candidate variants.**
(XLS)

Table S4 **667 pathogenic candidates regardless of gene ontology.**
(XLS)

Table S5 **Statistical results of 26 candidates in three different analyses.**
(XLS)
