# Supplementary material for: Exomic Sequencing of Immune-Related Genes Reveals Novel Candidate Variants Associated with Alopecia Universalis
Source: PLoS One. 2013 Jan 11;8(1):e53613. doi: 10.1371/journal.pone.0053613 (PMC3543254; doi:10.1371/journal.pone.0053613)
Supplement: Figure S1 — The flow of filtration steps used for candidate variant selection. (PPTX) [file pone.0053613.s001.pptx]

## Slide 1
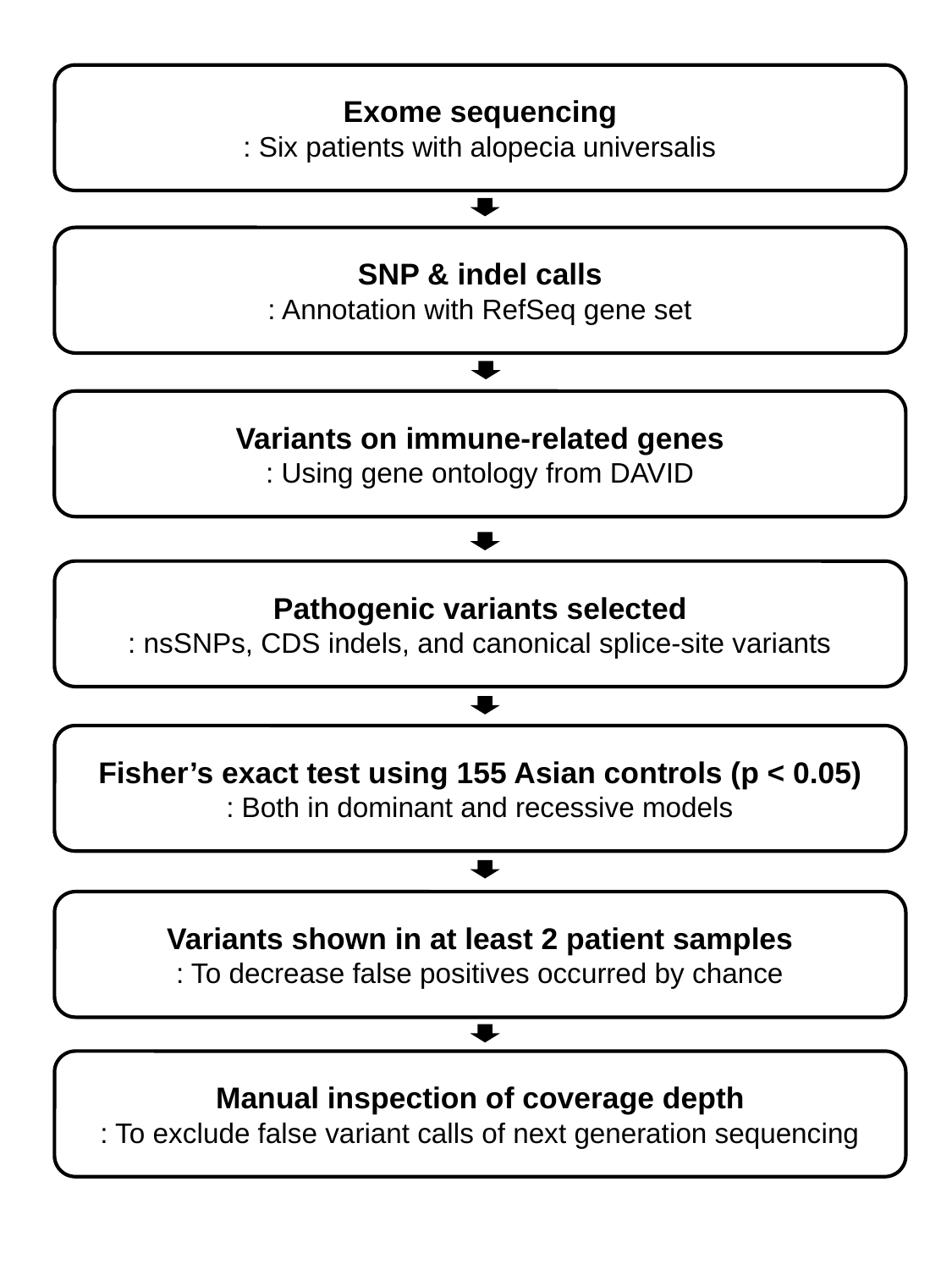

Exome sequencing
: Six patients with alopecia universalis
SNP & indel calls
: Annotation with RefSeq gene set
Variants on immune-related genes
: Using gene ontology from DAVID
Pathogenic variants selected
: nsSNPs, CDS indels, and canonical splice-site variants
Fisher’s exact test using 155 Asian controls (p < 0.05)
: Both in dominant and recessive models
Variants shown in at least 2 patient samples
: To decrease false positives occurred by chance
Manual inspection of coverage depth
: To exclude false variant calls of next generation sequencing
