# Supplementary material for: Exomic Sequencing of Immune-Related Genes Reveals Novel Candidate Variants Associated with Alopecia Universalis
Source: PLoS One. 2013 Jan 11;8(1):e53613. doi: 10.1371/journal.pone.0053613 (PMC3543254; doi:10.1371/journal.pone.0053613)
Supplement: Table S1 — Demographic data and clinical presentations of study subjects. (DOC) [file pone.0053613.s002.doc]

**Table S1. Demographic data and clinical presentations of study subjects**

| **Patient  no.** | **Age, years** | **Gender** | **Onset-age, years** | **Disease duration, years** | **Family history of AA** | **Body hair involvement** | **Nail dystrophy** | | **Comorbid disorders** |
| --- | --- | --- | --- | --- | --- | --- | --- | --- | --- |
| **1** | 4 | F | 9mo | 3 | - | eyebrow | + | | Atopic dermatitis |
| **2** | 24 | M | 10 | 14 | - | eyebrow, pubic, axilla | + | | - |
| **3** | 7 | M | 15mo | 5 | - | eyebrow | + | | Atopic dermatitis |
| **4** | 12 | F | 9 | 3 | + | eyebrow | - | | Thyroid disease Allergic rhinitis |
| **5** | 4 | F | 1 | 3 | + | eyebrow | - | | - |
| **6** | 23 | M | 5 | 18 | + | eyebrow, pubic, axilla | - | | - |
| **7** | 3 | M | 1 | 2 | - | eyebrow | - | | - |
| **8** | 12 | M | 3 | 9 | + | eyebrow | + | | - |
| **9** | 12 | M | 3 | 9 | + | eyebrow | + | | - |
| **10** | 29 | M | 26 | 3 | - | eyebrow, pubic, axilla | - | | - |
| **11** | 23 | F | 19 | 4 | - | eyebrow, pubic, axilla | - | | Psoriasis |
| **12** | 33 | M | 30 | 3 | + | eyebrow, pubic, axilla | - | | - |
| **13** | 43 | M | 38 | 5 | - | eyebrow, pubic, axilla | - | | - |
| **14** | 24 | M | 12 | 12 | - | eyebrow, pubic, axilla | - | | - |
| **15** | 22 | F | 18 | 4 | - | eyebrow, pubic, axilla | - | | - |
| **16** | 8 | M | 5 | 3 | - | eyebrow | - | | - |
| **17** | 3 | F | 9mo | 1 | - | eyebrow | + | | Thyroid disease |
| **18** | 4 | F | 3 | 1 | - | eyebrow | - | - | |
| **19** | 7 | M | 4 | 3 | - | eyebrow | - | - | |
| **20** | 9 | M | 5 | 4 | - | eyebrow | - | - | |

AA, alopecia areata; mo, months.
